# Supplementary material for: DET1-mediated degradation of a SAGA-like deubiquitination module controls H2Bub homeostasis
Source: eLife. 2018 Sep 7;7:e37892. doi: 10.7554/eLife.37892 (PMC6128693; doi:10.7554/eLife.37892)
Supplement: Figure 5—source data 1. [file elife-37892-fig5-data1.pptx]

## Slide 1
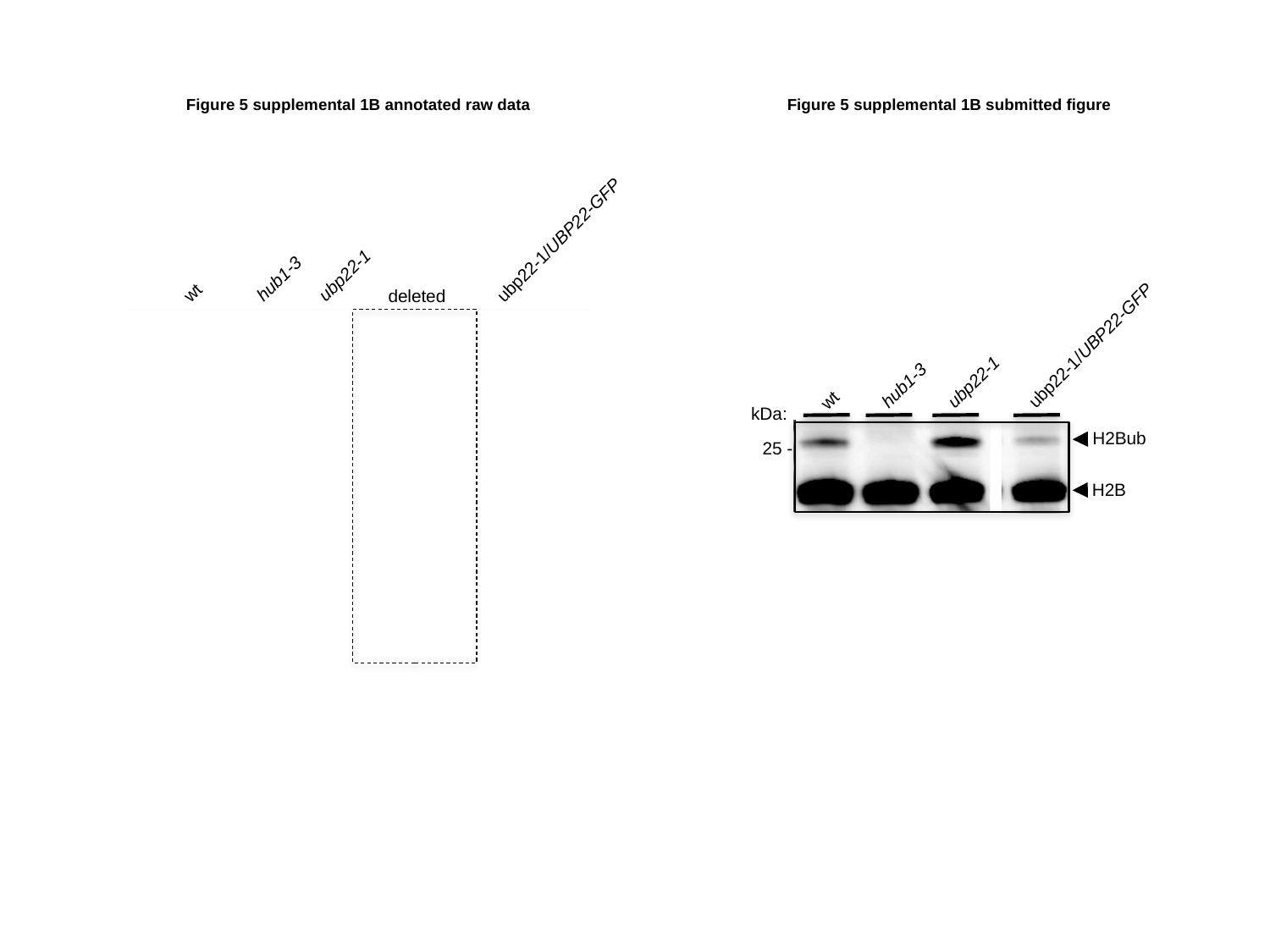

Figure 5 supplemental 1B annotated raw data
Figure 5 supplemental 1B submitted figure
ubp22-1/UBP22-GFP
ubp22-1
hub1-3
wt
deleted
ubp22-1/UBP22-GFP
ubp22-1
hub1-3
wt
kDa:
H2Bub
25 -
H2B
